# Evaluation of selective attention in patients with misophonia^☆^

**DOI:** 10.1016/j.bjorl.2018.02.005

**Published:** 2018-03-21

**Authors:** Fúlvia Eduarda da Silva, Tanit Ganz Sanchez

**Affiliations:** a Universidade de São Paulo (USP), Pós-Graduação em Ciências, São Paulo, SP, Brazil; b Instituto Ganz Sanchez, São Paulo, SP, Brazil; c Universidade de São Paulo (USP), Faculdade de Medicina, Departamento de Otorrinolaringologia, São Paulo, SP, Brazil

**Keywords:** Misophonia, Tinnitus, Hyperacusis, Selective attention, Auditory processing, Misofonia, Zumbido, Hiperacusia, Atenção seletiva, Processamento auditivo

## Abstract

**Introduction:**

Misophonia is characterized by the aversion to very selective sounds, which evoke a strong emotional reaction. It has been inferred that misophonia, as well as tinnitus, is associated with hyperconnectivity between auditory and limbic systems. Individuals with bothersome tinnitus may have selective attention impairment, but it has not been demonstrated in case of misophonia yet.

**Objective:**

To characterize a sample of misophonic subjects and compare it with two control groups, one with tinnitus individuals (without misophonia) and the other with asymptomatic individuals (without misophonia and without tinnitus), regarding the selective attention.

**Methods:**

We evaluated 40 normal-hearing participants: 10 with misophonia, 10 with tinnitus (without misophonia) and 20 without tinnitus and without misophonia. In order to evaluate the selective attention, the dichotic sentence identification test was applied in three situations: firstly, the Brazilian Portuguese test was applied. Then, the same test was applied, combined with two competitive sounds: chewing sound (representing a sound that commonly triggers misophonia), and white noise (representing a common type of tinnitus which causes discomfort to patients).

**Results:**

The dichotic sentence identification test with chewing sound, showed that the average of correct responses differed between misophonia and without tinnitus and without misophonia (*p* = 0.027) and between misophonia and tinnitus (without misophonia) (*p* = 0.002), in both cases lower in misophonia. Both, the dichotic sentence identification test alone, and with white noise, failed to show differences in the average of correct responses among the three groups (*p* ≥ 0.452).

**Conclusion:**

The misophonia participants presented a lower percentage of correct responses in the dichotic sentence identification test with chewing sound; suggesting that individuals with misophonia may have selective attention impairment when they are exposed to sounds that trigger this condition.

## Resumo

**Introdução:**

A misofonia é caracterizada pela aversão a certos sons muito seletivos, que evocam uma forte reação emocional. Tem sido demonstrado que a misofonia, bem como o zumbido, está associada à hiperconectividade entre os sistemas auditivo e límbico. Indivíduos com zumbido incômodo podem ter comprometimento da atenção seletiva, mas isso ainda não foi demonstrado em caso de misofonia.

**Objetivo:**

Caracterizar uma amostra de indivíduos misofônicos e compará-la com dois grupos controle, um com indivíduos com zumbido (sem misofonia) e o outro com indivíduos assintomáticos (sem misofonia e sem zumbido) em relação à atenção seletiva.

**Método:**

Avaliamos 40 participantes com audição normal: 10 com misofonia, 10 com zumbido (sem misofonia) e 20 sem zumbido e sem misofonia. Para avaliar a atenção seletiva, o teste de identificação de sentenças dicóticas foi usado em três situações: no primeiro, aplicou-se o teste em português do Brasil. Então, o mesmo teste foi aplicado em duas outras situações, combinado com dois sons competitivos: som de mastigação (representa um som que geralmente desencadeia misofonia) e ruído branco (representa um tipo comum de zumbido que causa desconforto nos pacientes).

**Resultados:**

No teste de identificação de sentenças dicóticas com som de mastigação, observou-se que a média de respostas corretas diferiu entre os grupos misofonia e sem zumbido e sem misofonia (*p* = 0,027) e entre misofonia e zumbido (sem misofonia) (*p* = 0,002) e, em ambos os casos, foi menor em misofonia. Em relação ao teste feito isoladamente e com ruído branco, nenhuma diferença foi observada na média das respostas corretas nos três grupos (*p* ≥ 0,452).

**Conclusão:**

Os participantes do grupo misofonia apresentaram uma menor porcentagem de respostas corretas no teste de identificação de sentenças dicóticas com som de mastigação, sugeriu que indivíduos com misofonia podem ter comprometimento da atenção seletiva quando expostos a sons que desencadeiam a condição.

## Introduction

Misophonia is characterized by the aversion to very selective sounds, which are usually of low level, repetitive and that provoke a strong, abrupt and disproportionate emotional reaction. It has been proposed that misophonia presents some general similarities with tinnitus,^1^ which is an internal sound, perceived in the ears or inside the head, without being produced by an external source.^2^ It was proposed that misophonia and tinnitus are both associated with hyperconnectivity between the auditory and limbic systems,^3^ suggesting that both would evoke exacerbated reactions to the respective internal (tinnitus) or external (misophonia) sounds.^1^

Considering the similarities between misophonia and tinnitus, one of the main complaints of these patients is the difficulty to disconnect their attention from the sounds (internal or external) during bedtime or during daily tasks, what justifies their impairment in quality of life. In fact, impaired selective attention has been demonstrated in patients with bothersome tinnitus,^4^, ^5^ but not yet in those with misophonia.

The Dichotic Sentence Identification (DSI) Test, adapted to Brazilian Portuguese, is one of the good tools to evaluate the selective attention.^6^ This test is divided into six bands, including the evaluation of binaural integration and directed report on the attention to the right or to the left ears. During the consecutive steps of the test, two out of ten different sentences are simultaneously presented in each ear.

We consider the hypothesis that individuals with misophonia, as already demonstrated for those with tinnitus, may present impairment of selective attention. Thus, the aim of this study was to evaluate the selective auditory attention in a sample of individuals with misophonia and two control groups, one with tinnitus (without misophonia) and the other one asymptomatic (without tinnitus and without misophonia).

## Methods

### Ethics and study design

This cross-sectional research was approved by Research Ethics Committee (n° 1458/15) and was performed at a reference center for patients with tinnitus and/or misophonia.

The participants were pre selected from announcements containing the goals of the research which were posted in the social media platform (Facebook) and/or emailed to a mailing list of subjects. All the ones who were recruited signed the Free Informed Consent Form.

### Participants and inclusion criteria

The sample of this study was comprised of three groups: the Misophonia Group (MG, *n* = 10), the Tinnitus Control Group (TCG, *n* = 10) and the Asymptomatic Control Group (ACG, *n* = 20).

The following inclusion criteria were established for all groups: (a) Normal bilateral hearing thresholds, stated as equal to or less than 25 dBHL at all frequencies from 250 to 8000 Hz in both ears; (b) Age above 12 years old of both genders; (c) Fluent reading ability regardless of educational status.

For specific groups, further criteria were established: (a) For MG: clinical history of misophonia with a score of annoyance equal to or greater than 6 in the Visual Analogue Scale (VAS) from 0 to 10; (b) For the TCG: absence of clinical history of misophonia and presence of continuous tinnitus perception with a score of annoyance equal to or greater than 6 in the Visual Analog Scale; (c) For the ACG: absence of clinical history of misophonia and tinnitus.

In order to fulfill the first inclusion criterion described above, a total of 43 subjects had their external auditory canal and tympanic membrane inspected with a Welch Allyn otoscope (model 19090) and their hearing thresholds assessed by the calibrated audiometer Madson Astera II and Sennheiser HAD 200 audiometric headphones. Three individuals of the TCG were excluded for having abnormal hearing thresholds in one or more frequencies and were replaced by the next three volunteers on the waiting list.

Thus, the final sample of the study included subjects with the following characteristics:

1) MG (*n* = 10): 6 females and 4 males, average age 31.6 years, median age 31.5 years.
2) TCG (*n* = 10): 6 females and 4 males, average age 42.2 years, median age 40.5 years.
3) ACG (*n* = 20): 12 females and 8 males, average age 31.7 years, median age 30.0 years.

### Procedures

The selective attention was evaluated by the Brazilian Portuguese version of the Dichotic Sentence Identification (DSI) test, which allows assessing the auditory capacity of figure-background for verbal sounds (identifying speech sounds in the presence of other speech sounds). The verbal sound recognition in dichotic listening acts as the underlying auditory physiological mechanism.^6^

The DSI test is comprised of six stages: calibration (Stage 1), training (Stage 2), binaural integration (Stage 3), directed report on the right ear (Stage 4), directed report on the left ear (Stage 5) and training (Stage 6). It consists of ten sentences, and two of them are presented simultaneously in each ear.

In order to adapt this conventional version of DSI to the needs of this study, we imported the Stages 1 to 5 of the test (Stage 6 was excluded because it is targeted for therapy) into the Audacity Software, which allows edition, importation and exportation of different formats of audio files. Two distracting sounds were imported into Audacity Software: (1) A white noise, which represents a common type of tinnitus that causes discomfort in patients; (2) A chewing sound, which represents a sound that commonly triggers misophonia. To assure the good quality of these sounds, both were edited, treated and mixed by a specialized professional who subsequently looped them in order to be presented together with the conventional version of DSI and to remain stable during the presentation.

The conventional DSI was applied in a way that each subject was instructed to read the sentences in a table to become familiar with the stimuli. After the calibration (Stage 1), the test was applied at 50 dBNA and involved the identification of the sentences, with visual support for responses. At Stages 2 and 3 (training and binaural integration), the individual was asked to indicate both sentences heard in the visual support; at Stages 4 and 5 (directed report to the right and to the left), the subject should indicate in the visual support only the sentence provided to the ear under assessment.^7^

After applying the test as described above, the Stages 3–5 of (DSI) test were repeated twice, adding one of the two distracting sounds elaborated in the Audacity Software at a time (white noise or chewing sound), with signal-to-noise ratio equal to 15 dBNA. To define the initial order of the distracting sound to be presented, subjects were randomized to 1:1.

## Results

Considering that all participants of the three groups (MG = 10; TCG = 10, ACG = 20) underwent three different situations (DSI test in a conventional way, with white noise and with chewing sound in randomized order of presentation), such individuals have been evaluated for the percentage of correct response when exposed to the different stages of each situation: binaural integration (right and left ears) and directed report (right or left ear).

The analysis of the gender distribution showed that there was a slight predominance of females in all groups (6/10 in MG and in TCG; 12/20 in ACG), which made them homogeneous. Regarding the age distribution, Table 1 shows the *p*-value of the equality of the average ages using the Welch test in the three groups, considering different variances. At the significance level of 5%, as applied to biological studies, there is evidence that the averages are not equal (*p* = 0.037). So, Table 2 shows the *p*-values of the *t*-student test to compare such averages, two by two (Games-Howell's method for multiple comparisons considering different variances). There was difference only between the average age of the TCG and ACG, which was higher in TCG, but not between TCG and MG, probably because ACG had a bigger sample.^8^

**Table 1:** Ages of the participants of MG, TCG and ACG and the *p*-value of the equality of the average ages.

Group	*N*	Mean	SE	Minimum	Median	Maximum	*p*-value
MG	10	31.60	9.20	14	31.5	48	0.037
TCG	10	42.20	10.72	29	40.5	59	
ACG	20	31.75	4.96	25	30.0	45	

*N*, sample size; SE, standard error.

**Table 2:** *p*-values of the *t*-student test to compare the average ages, two by two.

Comparison	*p*-value
Mean_MG_–Mean_ACG_	0.999
Mean_TCG_–Mean_ACG_	0.034
Mean_TCG_–Mean_MG_	0.072

Table 3, Table 4, Table 5 describe the percentage of correct responses for the MG, TCG and ACG groups, respectively, considering the stages of (DSI) test and situation. It is possible to observe the absence of variation or very small standard deviations in directed report stages of the three groups, where the percentages of correct responses are close or equal to 100%. It is also noted that the mean or median of correct response in the right ear is greater than or equal to the left ear for all groups and situations. In general, the mean or median of the percentage of correct responses that involves the right ear was greater than or equal to the mean or median of the percentage of correct responses involving the left ear, for all groups and situations.

**Table 3:** Descriptive statistic of the percentage (%) of correct responses for MG (*n* = 10) in stage and situation.

Situation	Stage	Mean	SD	Minimum	Median	Maximum
(DSI) test	Binaural integration R	94	6.99	80	95	100
	Binaural integration L	94	6.99	80	95	100
	Directed report R	100	0.00	100	100	100
	Directed report L	99	3.16	90	100	100
(DSI) test with white noise	Binaural integration R	96	6.99	80	100	100
	Binaural integration L	92	9.19	80	95	100
	Directed report R	89	31.43	0	100	100
	Directed report L	99	3.16	90	100	100
(DSI) test with chewing	Binaural integration R	91	5.68	80	90	100
	Binaural integration L	81	21.32	30	85	100
	Directed report R	98	4.22	90	100	100
	Directed report L	86	27.57	10	95	100

SD, standard deviation; R, right; L, left; *n*, sample number.

**Table 4:** Descriptive statistic of the percentage (%) of correct responses for TCG (*n* = 10) in stage and situation.

Situation	Stage	Mean	SD	Minimum	Median	Maximum
(DSI) test	Binaural integration R	93	8.23	80	95	100
	Binaural integration L	91	8.76	80	90	100
	Directed report R	98	4.22	90	100	100
	Directed report L	99	3.16	90	100	100
(DSI) test with white noise	Binaural integration R	95	7.07	80	100	100
	Binaural integration L	89	12.87	70	95	100
	Directed report R	100	0.00	100	100	100
	Directed report L	100	0.00	100	100	100
(DSI) test with chewing	Binaural integration R	97	6.75	80	100	100
	Binaural integration L	92	7.89	80	90	100
	Directed report R	100	0.00	100	100	100
	Directed report L	100	0.00	100	100	100

SD, standard deviation; R, right; L, left; *n*, sample number.

**Table 5:** Descriptive statistic of the percentage (%) of correct responses for ACG (*n* = 20) in stage and situation.

Situation	Stage	Mean	SD	Minimum	Median	Maximum
(DSI) test	Binaural integration R	93	8.65	70	95	100
	Binaural integration L	92	9.33	80	95	100
	Directed report R	99	3.08	90	100	100
	Directed report L	97	6.57	80	100	100
(DSI) test with white noise	Binaural integration R	94	7.45	80	95	100
	Binaural integration L	86	10.95	60	90	100
	Directed report R	100	2.24	90	100	100
	Directed report L	100	0.00	100	100	100
(DSI) test with chewing	Binaural integration R	92	8.34	80	90	100
	Binaural integration L	86	15.69	50	90	100
	Directed report R	100	2.24	90	100	100
	Directed report L	100	3.08	90	100	100

SD, standard deviation; R, right; L, left; *n*, sample number.

**Figure 1:**
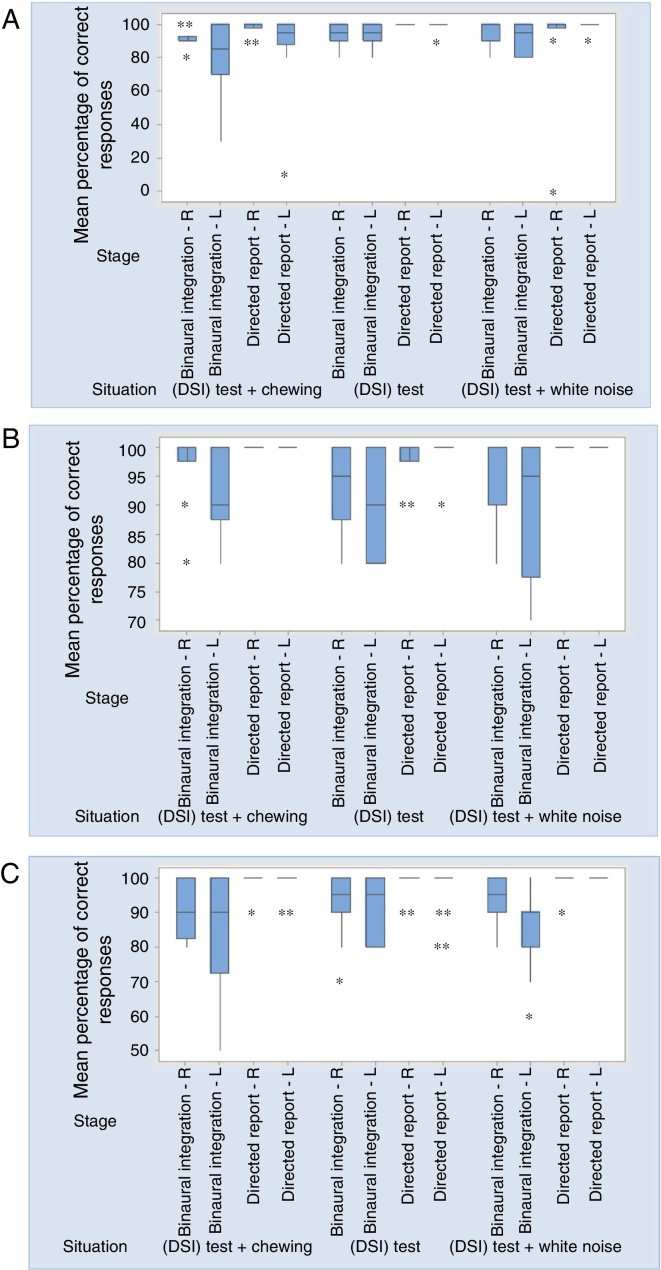
(A, B, C) Boxplots of the percentage of correct response for stages and situation in MG, TCG and ACG, respectively.

Misophonia Group showed very low minimum values in the (DSI) test with white noise in the directed report (right ear) and in the (DSI) test with chewing sound in the binaural integration (left ear) and directed report (left ear) stages. Fig. 1 shows the individual values of the percentage of correct responses for group, situation and stage. Three values are much lower than the others, which represent percentages of correct responses of the same individual.

In order to analyze the variable percentage of correct responses, a model of ANOVA with repeated measurements and three fixed factors (group, stage and situation) was adjusted because all individuals were evaluated under three situations in all stages.^8^, ^9^ The effect of the individuals was considered as random, and the Stage and Situation as repeated measures. The significance level adopted for all statistical hypotheses was 0.05 (5%).

**Figure 2:**
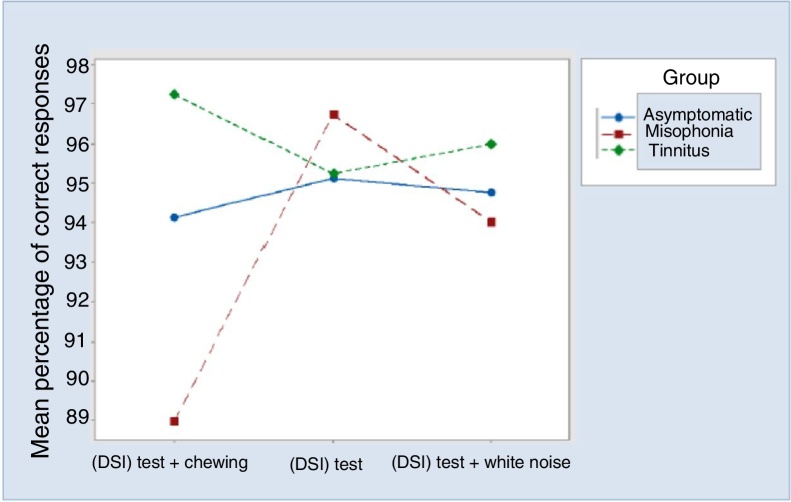
Mean percentage of correct responses for group and situation.

Waste charts were constructed^10^ and confirmed that the model was well adjusted. There was no evidence of an interaction effect between the stage and the other factors (*p* ≥ 0.094). However, there is evidence (*p* ≥ 0.094) of interaction effect between group and situation (Fig. 2).

**Figure 3:**
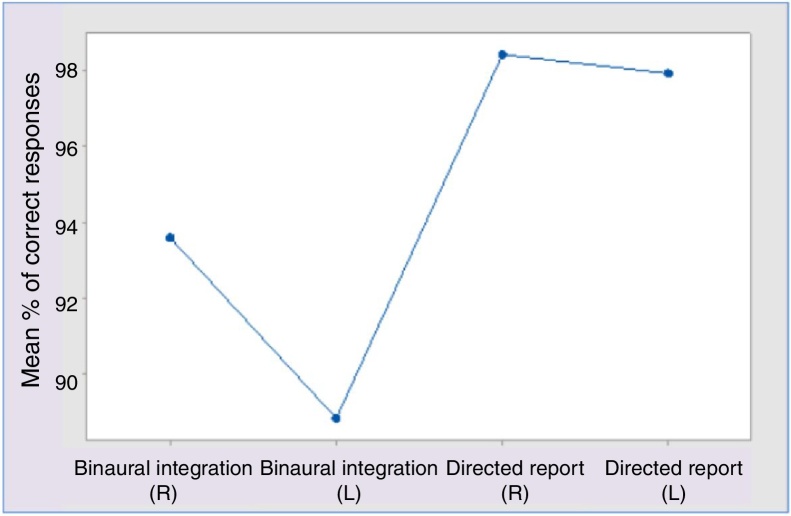
Mean percentage of correct response for stage.

Statistically significant difference occurred in the following analysis: (1) In the binaural integration stage, the average of correct responses in the right ear was greater than in the left ear (*p* < 0.001); (2) The average of correct responses in each ear in the directed report stage was greater than in the binaural integration stage, in both right and left ear (*p* < 0.001). In the binaural integration stage, the three groups presented a lower performance when compared to the directed report stage (Fig. 3).

## Discussion

At first, we had the intention of enrolling individuals with the same gender and age distribution for the three studied groups. However, data analysis showed that the TCG participants had higher average than the other two groups. A possible justification is that the prevalence of tinnitus increases proportionately with age,^11^ reaching 33% of elderly individuals.^12^ However, even with the statistical difference in relation to the other groups, the average and median ages of the TCG participants were less than 45 years, showing that no elderly was included. Therefore, we believe that this difference among the adult participants has not been relevant considering the aims of this study.

The association between misophonia and tinnitus has already been reported in the literature. A previous study^13^ found that among 149 patients with tinnitus and hyperacusis, 57% also had misophonia. In addition, in a sample of patients with tinnitus and normal hearing, 10% also presented misophonia.^14^ In our study, some patients with misophonia also complained of tinnitus. However, the latter has always been considered by patients as a secondary symptom, and for this reason, they have not been excluded from the study.

Although attention is a topic that has not been approached in misophonia yet, there is some evidence supporting the claim that tinnitus severity compromises the sustained attention.^15^, ^16^, ^17^ Tinnitus may be caused by aberrant engagement of top-down attention, or abnormal bottom-up attention, wherein internal noise gains salience when the external environment is quiet. It may also be an interaction of the two processes.^18^ Moreover, interactions between top-down and bottom-up processes contribute to the allocation of limited perceptual processing resources to one or more sound-parameter dimensions.^19^ Presumably, top-down and bottom-up attention processes can share neural resources, although their expressions in brain networks may depend on the specific types of tasks stimuli and the behavioral and cognitive performance requirements of the task procedure.^18^

Based on this information, the idea that patients with misophonia may suffer from selective attention impairment seems to be coherent. We chose to access the selective attention of individuals with misophonia through the Brazilian Portuguese version of Dichotic Sentence Identification test combined with two uncomfortable and distracting sounds: white noise (a common type of annoying tinnitus) and chewing sound (a common sound that annoys misophonic subjects). We expected to identify impairment of selective attention only in the presence of these sounds.

During the application of the (DSI) test with chewing, some MG participants spontaneously reported tachycardia, sweating, nervousness and anxiety at the beginning. However, they gradually adapted to that uncomfortable sound produced by the computer. These participants affirmed that if the same sound was produced by a human close to them in real life, probably it would make the performance of the tests more difficult or even impossible to conclude. In turn, this data may justify the slightly different results of the statistical analysis. For example, in the analysis of each group, the mean percentage of correct responses remained stable in the Misophonia Group in all of three situations (DSI test, DSI test with white noise and DSI test with chewing sound). Only one participant obtained a discrepant performance in the (DSI) test with chewing sound, for binaural integration (left ear) and left directed report stages. This participant attributed score 10 for the discomfort of misophonia on the visual analogue scale. In addition, only this participant reported that his own sounds also bothered him. Considering that this individual perfectly fitted the selection criteria and that the trigger sounds may influence individuals in a heterogeneous way in the real life, we chose not to exclude him from the study.

The analysis of the situations showed that, in the (DSI) test with chewing, the participants of MG presented a lower mean of correct responses than TCG (*p* = 0.002) and ACG (*p* = 0.027), which have not occurred in the (DSI) test and (DSI) test with white noise. At first, these results seemed to highly correspond to the clinical routine of attending patients with misophonia, when they strongly complain of their attention being selectively disturbed by specific annoying sounds, but not by others. TCG and ACG patients maintained the scores of correct responses established in the three situations. These results suggest that the presence of an external sound, similar or not to tinnitus, have not provoked selective attention impairment. These findings of TCG do not correspond to the clinical observation that tinnitus patients complain of their performance in daily tasks when they hear their own tinnitus or sounds similar to it.

It was interesting to notice that the average of correct responses in the right ear was significantly greater than in the left ear (*p* < 0.001) in all stages of the tests for the three groups. The difference in performance between the right and left ears in the administration of dichotic listening tests may occur because of the influence of the brainstem nuclei in the efferent regulation of other high cortical structures. During the dichotic presentation of a speech signals, the ones directed for the non-dominant hemisphere are partially degraded by the circuits of the dominant hemisphere,^20^ thus demonstrating the existing functional hemispheric differences.^21^

Moreover, the average of correct responses in each ear in the directed report stage was greater than in the binaural integration stage, in both right and left ear (*p* < 0.001) for the three groups. According to Costa-Ferreira et al.,^22^ the difficulties observed in the binaural integration stage, in dichotic listening tests, may occur because of the alterations in the skills of attention and/or working memory.^22^

Although this study has contributed to the understanding of selective attention in patients with misophonia, a few limitations need to be surpassed in future studies: (1) The relative small sample of patients with tinnitus and normal hearing thresholds – established as inclusion criteria for TCG – was hard to find, and we opted to pair the number of participants in TCG and MG. We assume that a greater sample would have been able to show more participants with lower percentages of correct responses in the tests about selective attention; (2) After the beginning of the tests, we noticed by the spontaneous opinion of some participants of MG that the distracting sounds produced by a computer may have provoked a lower impact on selective attention than expected in real life. Other methodologies, such as the use of functional imaging for mapping the activation of the brain areas before, during and after the presence of trigger sounds, would collaborate to further understanding the selective attention in patients with misophonia and the development of specific strategies to reduce the discomfort in these individuals.

## Conclusions

The participants of Misophonia Group presented a lower percentage of correct responses in the Dichotic Sentence Identification (DSI) test presented with and additional chewing sound when compared to the conventional presentation of the (DSI) test and with the presentation of an additional sound of white noise. Participants of both control groups (TCG, with tinnitus, and ACG, without tinnitus and misophonia) have not presented a significant difference in the average of correct responses in such tests. Thus, we conclude that the selective attention may be impaired in patients with misophonia when they are exposed to sounds similar to those that trigger their annoyance in daily life.

## Conflicts of interest

The authors declare no conflicts of interest.
